# Sustaining HIV prevention success in Australia through person‐centred approaches

**DOI:** 10.1002/jia2.70007

**Published:** 2025-10-08

**Authors:** Benjamin R. Bavinton, James Gray, Andrew E. Grulich

**Affiliations:** ^1^ The Kirby Institute UNSW Sydney Sydney New South Wales Australia; ^2^ Health Equity Matters Sydney New South Wales Australia

1

Person‐centred care is a critical element of HIV care. Global and country‐level consensus statements, including from Australia, have emphasized holistic, rights‐based approaches centring the autonomy, dignity, experiences, diverse needs, preferences and wellbeing of people living with HIV (PLHIV) [1]. However, the focus has been on HIV care with less focus on person‐centred prevention, despite its recent integration into the Joint United Nations Programme on HIV/AIDS (UNAIDS) goal that 95% of individuals at risk of HIV will utilize “appropriate, person‐centred, prioritised, and effective combination prevention options” by 2025 [2].

Drawing on the concept of person‐centred care, person‐centred HIV prevention [3] prioritizes individuals – their autonomy, dignity, rights, decisions and experiences – over interventions or risk categories. It recognizes that individuals are best placed to determine suitable prevention methods, respecting their personal choice and agency. This approach acknowledges the dynamic nature of needs and choices, shaped by personal, contextual and structural factors, such as stigma, discrimination, criminalization and socio‐economic conditions. It requires services to be appropriate, responsive and accessible, particularly for marginalized communities facing barriers to care.

Australia has achieved considerable success in HIV prevention, and has an ambitious goal to virtually eliminate HIV transmission by 2030 [4]. In gay, bisexual and other men who have sex with men (GBMSM) in certain urban areas, reductions in HIV diagnoses are approaching the UNAIDS 2030 goal of a 90% reduction from a 2010 baseline [5]. Nonetheless, disparities are evident, particularly among overseas‐born GBMSM and those residing outside inner‐city suburbs. While nationwide HIV diagnoses decreased by 54% in Australian‐born GBMSM between 2010 and 2023, there was a 55% increase in migrant GBMSM, and by 2023, 59% of all GBMSM diagnoses were in migrants [6]. Diagnoses among sex workers and people who use drugs are very low, and HIV rates are also very low among heterosexuals, though those born overseas are at higher risk [6].

Community and community‐based organizations (CBOs) have long been integral to HIV prevention, and play an essential role in understanding, articulating and advocating for the needs and preferences of communities affected by HIV [7]. Referred to in Australia as the “partnership approach” [8], collaboration between community, government, policymakers, clinicians and researchers has ensured that communities affected by HIV are key players in decision‐making. Despite occasional fluctuations, there has been sustained investment in Australia's HIV‐focused CBOs, including support to diversify their remit to encompass broader elements of LGBTQ+ health, other blood‐borne viruses and/or sexually transmitted infections (STIs).

Australian CBOs have been instrumental in delivering peer‐led, sex‐positive, inclusive and pragmatic HIV prevention health promotion, peer education and social marketing. Indeed, the first condom use campaign in Australia was produced and delivered by and for gay men within the community, even before many of the CBOs were formally established [9]. Government‐led HIV prevention social marketing is minimal in Australia, and CBOs predominantly deliver these campaigns. CBOs representing key populations such as GBMSM, sex workers and people who use drugs can be more responsive to community needs, have a greater understanding of effective messaging and can be more explicit in community‐centred, sex‐positive messaging than government agencies [7].

CBOs have also played a crucial role in service delivery, such as condom distribution, needle and syringe programmes, running community‐based HIV/STI testing sites (some of which were successful in delivering pre‐exposure prophylaxis [PrEP]) [10, 11] and scaling up HIV self‐testing via online platforms or vending machines. Peer navigation – often mentioned as a quintessential example of person‐centred care [1] – has been a vital component of supporting PLHIV. It has recently been recognized by the Australian Government as a potentially high‐impact tool to address barriers faced by migrants in HIV testing and prevention, with funding for a new national multicultural peer navigation project to be led by a CBO.

Australia has a publicly funded universal healthcare system providing free or subsidized primary healthcare. Integration of HIV testing and prevention into primary care exemplifies person‐centred principles and offers two major benefits: holistic care and patient choice. In many countries, HIV testing and PrEP are primarily offered in specialist HIV services and hospitals – a setup that may be effective for HIV care but is less likely to succeed in reaching the much larger populations needing access to prevention [12]. For prevention to be effective, it must be genuinely accessible, everywhere. From the inception of PrEP in Australia, any medical practitioner could prescribe it. This approach means that when a patient seeks HIV testing, STI testing or PrEP, they are attended to by a clinician capable of addressing more general health issues, such as mental health, sexual wellbeing and physical health. Specialist sexual health centres provide another choice for people's HIV prevention needs, with many centres having counselling teams and referral pathways to other specialist services.

However, further progress is necessary, and Australia must continue its long history of innovating and implementing person‐centred approaches. One example is the limited choice of PrEP options in Australia. Oral PrEP scale‐up led to rapid declines in HIV diagnoses and one of the highest per‐capita uptake rates globally [5, 12, 13], with community‐based surveys suggesting over three‐quarters of GBMSM at risk of HIV are taking PrEP [14]. However, essentially, only one PrEP product is widely available (oral tenofovir disoproxil* and emtricitabine [TD*/FTC]; although emtricitabine/tenofovir alafenamide can be legally ordered online and personally imported). Oral TD*/FTC is not suitable for everyone: some individuals have medical contraindications, while others experience side effects, dislike taking tablets, or struggle with adherence [15]. Decisions on government subsidy for new medicines in Australia are based on efficacy and cost‐effectiveness compared to current practice [16], meaning that the success of generic oral TD*/FTC PrEP – and its low cost – poses a challenge for the introduction of new PrEP products [17]. Despite long‐acting injectable Cabotegravir receiving early regulatory approval, a positive recommendation for government subsidy and ongoing advocacy from community organizations, price negotiations were unsuccessful, and this product is unavailable. In the meantime, choice is paramount in person‐centred care, and it is essential to enhance oral PrEP accessibility and affordability, especially for marginalized populations. Options being explored include nurse‐led PrEP provision at publicly funded sexual health centres, PrEP delivered by community pharmacists, extending the duration of PrEP prescriptions, telehealth PrEP services and research into peer‐provided PrEP [16].

To achieve national and global goals, Australia must build on its successes in prevention with a commitment to person‐centred principles – an approach that has long been embedded in the Australian response, even before the concept was formally articulated. Despite being in a context of universal healthcare and legal protections for sexual and gender minorities, disparities have emerged in the HIV epidemic. We must continue to innovate and implement person‐centred approaches to ensure all individuals have access to the prevention methods that are right for them.

## COMPETING INTERESTS

BRB has received research funding to his institution, and travel funding and honoraria from Gilead Sciences and ViiV Healthcare. AEG has received research funding to his institution from GSK and ViiV Healthcare, travel funding from ViiV Healthcare, and in‐kind research support from GSK.

## AUTHORS’ CONTRIBUTIONS

BRB conceptualized and drafted the manuscript. All authors reviewed and provided feedback on the manuscript.

## FUNDING

No funding was received for this manuscript. BRB is supported by a National Health and Medical Research Council (NHMRC) Investigator Grant (2027284). AEG is supported by an NHMRC Investigator Grant (2033249).

## Data Availability

Data sharing is not applicable to this article as no new data were created or analysed in this study.

## References

[1] ASHM , National Association of People With HIV Australia . Australian Consensus Statement on Person‐Centred HIV Care. Sydney: ASHM and NAPWHA; 2022.

[2] UNAIDS . End Inequalities. End AIDS. Global AIDS Strategy 2021–2026. Geneva: UNAIDS; 2021.

[3] Pantelic M , Stegling C , Shackleton S , Restoy E . Power to participants: a call for person‐centred HIV prevention services and research. J Int AIDS Soc. 2018;21:e25167. 30334609 10.1002/jia2.25167 PMC6193315

[4] Australian Government . Ninth National HIV Strategy 2024–2030. Canberra: Australian Government Department of Health and Aged Care; 2024.

[5] Keen P , Nigro SJ , Chan C , Bavinton BR , Aung HL , Holt M , et al. Progress towards the UNAIDS 2030 HIV prevention target in New South Wales, Australia: a population‐based study. Lancet Reg Health West Pac. 2025;57:101535. 40276649 10.1016/j.lanwpc.2025.101535 PMC12019848

[6] Kirby Institute . HIV, viral hepatitis and sexually transmissible infections in Australia: Annual Surveillance Report 2024. Sydney: Kirby Institute; 2024.

[7] Nous Group . Demonstrating the value of community control in Australia's HIV response: Final report for AFAO and Australia's State and Territory AIDS Councils. Sydney: Nous Group; 2016.

[8] Bernard D , Kippax S , Baxter D . Effective partnership and adequate investment underpin successful response: key factors in dealing with HIV increases. Sex Health. 2008;5(2):193–201. 18686337 10.1071/sh07078

[9] Power J . Movement, knowledge, emotion: gay activism and HIV/AIDS in Australia. Canberra: ANU E‐Press; 2011.

[10] Chan C , Patel P , Johnson K , Vaughan M , Price K , McNulty A , et al. Community‐based peer‐led HIV/sexually transmitted infection testing services in Sydney for gay and bisexual men captured an eighth of new HIV diagnoses in New South Wales, Australia. AIDS. 2021;35(11):1878–80. 34397488 10.1097/QAD.0000000000002982

[11] Schmidt H‐MA , McIver R , Houghton R , Selvey C , McNulty A , Varma R , et al. Nurse‐led pre‐exposure prophylaxis: a non‐traditional model to provide HIV prevention in a resource‐constrained, pragmatic clinical trial. Sex Health. 2018;15(6):595–7. 30257752 10.1071/SH18076

[12] Bavinton BR , Grulich AE . HIV pre‐exposure prophylaxis: scaling up for impact now and in the future. Lancet Public Health. 2021;6(7):e528‐e33. 34087117 10.1016/S2468-2667(21)00112-2

[13] Grulich AE , Guy R , Amin J , Jin F , Selvey C , Holden J , et al. Population‐level effectiveness of rapid, targeted, high‐coverage roll‐out of HIV pre‐exposure prophylaxis in men who have sex with men: the EPIC‐NSW prospective cohort study. Lancet HIV. 2018;5(11):e629–e37. 30343026 10.1016/S2352-3018(18)30215-7

[14] MacGibbon J , Chan C , Bavinton BR , Mao L , Smith AKJ , Molyneux A , et al. GBQ+ Community Periodic Survey: Sydney 2024. Sydney: Centre for Social Research in Health, UNSW Sydney; 2024.

[15] Haberer JE , Mujugira A , Mayer KH . The future of HIV pre‐exposure prophylaxis adherence: reducing barriers and increasing opportunities. Lancet HIV. 2023;10(6):e404–e11. 37178710 10.1016/S2352-3018(23)00079-6

[16] Arapali T , Warzywoda S , Smith AKJ , Keen P , Mac Phail C , Dowell‐Day A , et al. Innovative oral PrEP service delivery models and the policy, legal, and regulatory barriers to their implementation. Sydney: Kirby Institute, UNSW Sydney; 2024.

[17] Rui Z , Fairley CK , Cook A , Phanuphak N , He S , Tieosapjaroen W , et al. Optimizing HIV pre‐exposure prophylaxis and testing strategies in men who have sex with men in Australia, Thailand and China: a modelling study and cost‐effectiveness analysis. Lancet Glob Health. 2024;12(2):e243–e56. 38245115 10.1016/S2214-109X(23)00536-3

